# Patient Characteristics Associated With Preference for 480‐mL Oral Sodium Sulfate: A Prospective Clinical Study on Bowel Cleansing Efficacy and Taste Acceptability for Total Colonoscopy

**DOI:** 10.1002/deo2.70353

**Published:** 2026-05-19

**Authors:** Tomohiro Shimada, Taku Yamagata, Yoshihide Kanno, Takeshi Shimizu, Kai Korekawa, Hiroki Sato, Yutaka Eto, Yosei Sawai, Teruyuki Numata, Kei Ito

**Affiliations:** ^1^ Department of Gastroenterology Sendai City Medical Center Sendai Japan

**Keywords:** bowel preparation, colonoscopy, elderly, oral sulfate solution, patient preference

## Abstract

**Background and Aims:**

Oral sulfate solutions (OSSs) are used for low‐volume bowel preparation but have a distinctive bitter taste. This study evaluated the efficacy and tolerability of OSSs compared with polyethylene glycol plus ascorbate (PEG‐ASC) and identified patient factors associated with favorable acceptability.

**Methods:**

In this prospective single‐center study, patients undergoing colonoscopy using OSSs who had used PEG‐ASC for a previous colonoscopy within 2 years were enrolled. Cleansing quality (Boston Bowel Preparation Scale [BBPS]), ingested volume, adverse events, patient preference, and taste acceptability were assessed. Outcomes were also compared between elderly (≥75 years) and non‐elderly patients.

**Results:**

Among 263 patients, 98% achieved satisfactory cleansing (BBPS ≥ 6). Overall, 54% preferred OSS for their next procedure. Multivariate analysis identified favorable taste acceptability as the strongest predictor of preference (odds ratio [OR], 4.88). Favorable taste acceptability tended to be associated with male sex (OR, 1.58; *p* = 0.09) and age ≥ 75 years (OR, 1.69; *p* = 0.07). Older male patients showed significantly higher favorable taste acceptability than younger female patients (63% vs. 40%; *p* = 0.03). Elderly patients had lower total fluid intake (*p* = 0.04) and fewer adverse events (*p* < 0.01), particularly nausea, while maintaining cleansing quality equivalent to non‐elderly patients.

**Conclusions:**

OSS provided high‐quality cleansing with a reduced intake volume. Elderly patients tended to have favorable taste acceptability for OSS, and effective cleansing was achieved with even lower total fluid intake and fewer adverse events. OSS represents an advantageous bowel preparation option for the aging population.

**Trial Registration:**

UMIN‐CTR: UMIN000052571.

**Abbreviations**
ADR: adenoma detection rate
BBPS: Boston Bowel Preparation Scale
OR: odds ratio
OSS: oral sulfate solution
PEG: polyethylene glycol
PEG‐ASC: PEG plus ascorbate solution
RCT: randomized controlled trial
TCS: total colonoscopy
UMIN‐CTR: University Hospital Medical Information Network Clinical Trials Registry.

## 1 Introduction

Adequate bowel preparation for total colonoscopy (TCS) improves the adenoma detection rate (ADR) and reduces colorectal cancer mortality [1, 2, 3, 4]. Thus, the choice of bowel preparation agents is critical to the success of TCS. In Japan, polyethylene glycol (PEG)‐based solutions have been widely used [5]. Even with the improved PEG plus ascorbate solution (PEG‐ASC), patients must ingest up to 2 L of solution [6]. Insufficient intake may result in suboptimal cleansing, which can negatively affect ADR. Moreover, poor adherence to bowel preparation may reduce patient acceptance of TCS, ultimately lowering participation in colorectal cancer screening programs. These issues highlight the need for regimens that combine smaller volumes with effective cleansing.

Since 2021, a 480‐mL oral sulfate solution (OSS; Sulprep, Fuji Pharma Co., Ltd., Tokyo, Japan) has been commercially available in Japan. The maximum intake volume of OSS is 960 mL, which is considerably lower than that of PEG‐ASC. A Japanese randomized controlled trial (RCT) demonstrated that a smaller volume of an OSS was required compared to that of PEG‐ASC [5]. Furthermore, in a recent meta‐analysis, OSSs had a safety profile comparable to PEG, while providing superior cleansing quality and higher ADR [7]. However, the distinctive, bitter, concentrated lemon flavor of OSSs may compromise palatability, potentially affecting compliance. To date, this concern has not been systematically investigated.

Identifying patient characteristics associated with good acceptance of an OSS would enable clinicians to tailor bowel preparations, thereby improving compliance and ensuring adequate cleansing. The present study aimed to evaluate the cleansing efficacy of OSSs and to identify patient characteristics associated with favorable palatability, compared to previous experience with PEG‐ASC, the most commonly used agent in Japan.

## 2 Methods

### 2.1 Study Design

This was a single‐center, prospective, single‐arm observational study conducted at Sendai City Medical Center. The study protocol was approved by the institutional review board of our institution (approval No. 2023‐0060) in accordance with the Declaration of Helsinki. The study was prospectively registered with the University Hospital Medical Information Network Clinical Trials Registry (UMIN‐CTR; UMIN000052571).

### 2.2 Patients

Eligible patients were those who underwent TCS using an OSS and had previously undergone outpatient TCS with PEG‐ASC within the past 2 years. Participants were aged ≥18 years and provided written informed consent. Exclusion criteria included known taste disorders, inability to complete the questionnaire, allergies to OSSs, PEG, or sodium picosulfate, severe renal failure with or without hemodialysis, prior incomplete TCS, and the need for non‐standard preparation due to severe constipation.

### 2.3 Bowel Preparation

On the day before TCS, patients consumed a low‐residue diet and received 75 mg of sodium picosulfate at bedtime for both OSS and PEG‐ASC preparation.

For the OSS preparation, OSS was administered using a same‐day dosing regimen rather than a split‐dose protocol [5]. Three hours before the procedure, patients ingested 120 mL of an OSS, followed by 240 mL of water. This cycle was repeated until adequate cleansing was achieved, which was defined as the passage of clear or pale‐yellow liquid without particulate matter, based on patient or family assessment using standardized visual reference images provided before the procedure, up to a maximum OSS dose of 960 mL. If cleansing was inadequate after reaching this limit, PEG was administered as a rescue agent.

For the PEG‐ASC preparation used for previous TCSs, PEG‐ASC was administered with alternating intake of two cups of the solution and one cup of water. Preparatory measures and rescue agent use were essentially identical to the OSS regimen.

### 2.4 Questionnaire Survey

Immediately prior to TCS, patients completed a questionnaire assessing: (1) taste acceptability of the OSS compared with PEG‐ASC, rated on a 5‐point scale (excellent, good, neutral, poor, and very poor); (2) preferred preparation for future TCSs (OSS, PEG‐ASC, or either); and (3) adverse events during OSS ingestion (abdominal pain, nausea, bloating, vomiting). Taste responses of “excellent,” “good,” or “neutral” were defined as favorable taste acceptability.

### 2.5 Colonoscopy Procedure

TCSs were performed by physicians, including trainees, using a CF‐HQ290ZI or CF‐XZ1200I scope (Olympus Medical Systems, Tokyo, Japan) with an EVIS X1 video system. As an antispasmodic, either 20 mg of butylscopolamine or 1 mg of glucagon was administered intramuscularly or half doses intravenously. Sedation with midazolam or propofol was provided at the endoscopist's discretion, aiming for moderate to deep sedation according to the American Society of Anesthesiologists classification [8].

### 2.6 Data Collection

After the procedure, the endoscopist recorded sex, age, height, weight, indication, comorbidities, cecal or anastomotic intubation success, insertion and withdrawal times, OSS, and water volumes ingested, need for and volume of additional PEG, bowel cleansing quality assessed using the Boston Bowel Preparation Scale (BBPS) [9], and adverse events. BBPS scores ranged from 0 (unprepared) to 3 (excellent), assigned separately for the right, transverse, and left colon.

### 2.7 Outcome Measurement

Primary endpoints were bowel cleansing quality, ingested OSS and water volume, and adverse events. Secondary endpoints included taste acceptability of OSS compared with PEG‐ASC, as assessed by the questionnaire. In addition, patient characteristics associated with favorable taste acceptability were examined. Elderly patients were defined as those aged ≥75 years.

### 2.8 Statistical Analysis

Continuous variables were compared using the Mann–Whitney U test, and categorical variables using the chi‐square test or Yates’ correction. Logistic regression was used for univariate and multivariate analyses to identify independent factors associated with outcomes. Variables with *p* < 0.2 in univariate analysis were included in the multivariate model with age and sex as adjustment factors. Analyses were performed using EZR (Saitama Medical Center, Jichi Medical University, Saitama, Japan) [10]. A *p*‐value of <0.05 was considered statistically significant.

## 3 Results

**FIGURE 1:**
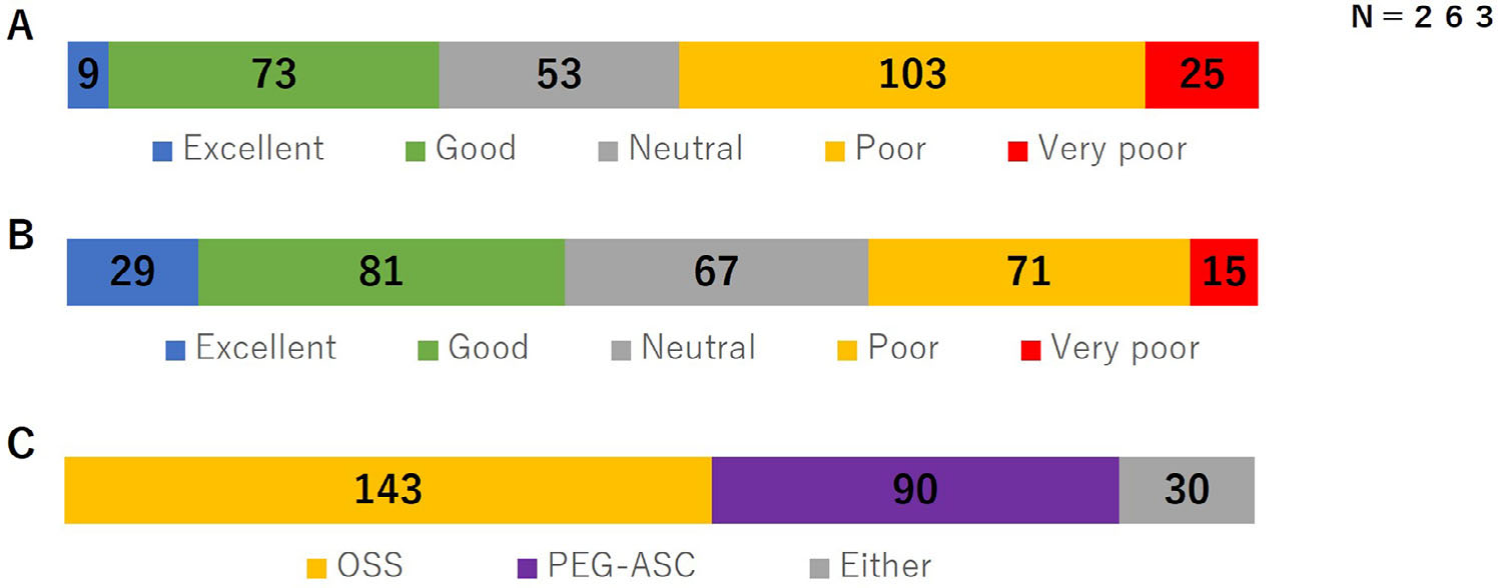
Patient questionnaire responses on taste, palatability, and preferred preparation for next TCS. (A) Taste. (B) Palatability. (C) Preferred preparation for next TCS. TCS: total colonoscopy, OSS: oral sulfate solution, PEG‐ASC: polyethylene glycol plus ascorbate.

**FIGURE 2:**
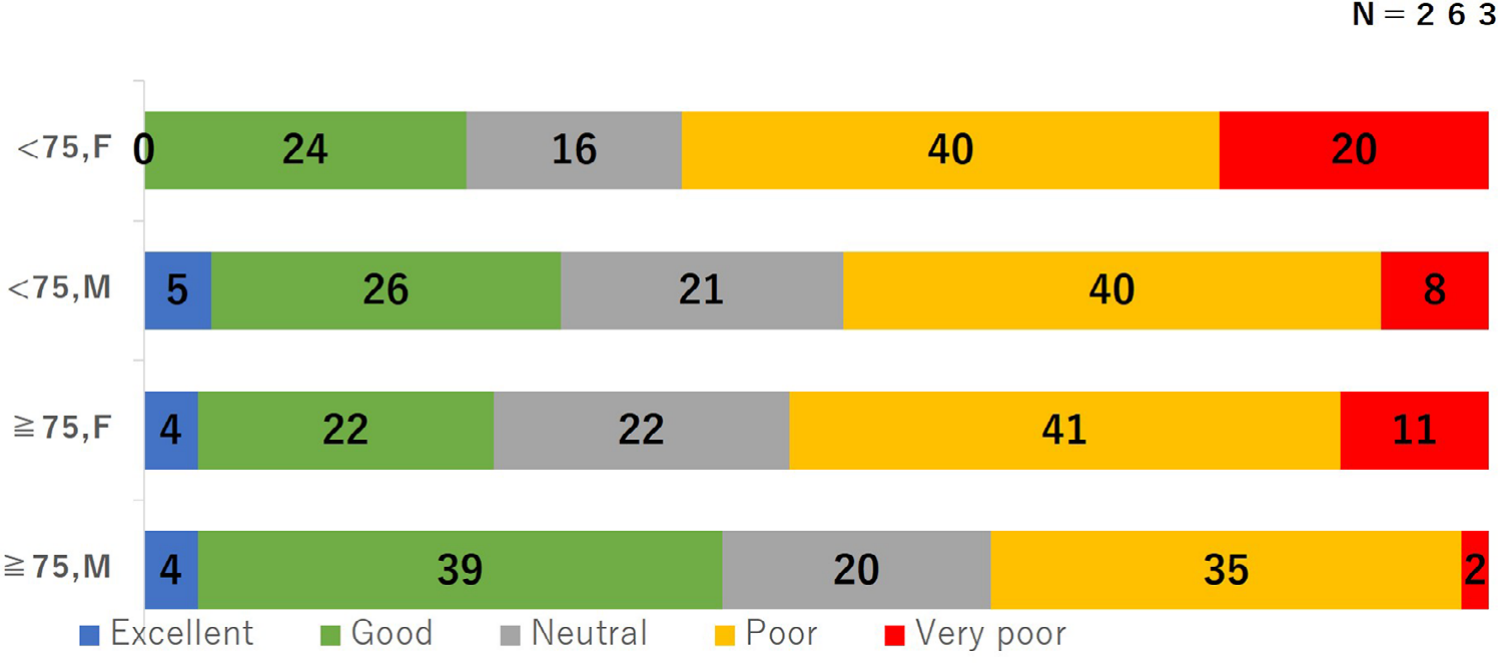
Taste acceptability of oral sulfate solution (OSS) stratified by age and sex. Proportion of patients who taste acceptability of oral sulfate solution, stratified by age and sex. Older male patients showed significantly higher acceptability than younger female patients (63% vs. 40%, *p* = 0.03).

Among 5746 TCS procedures performed during the study period, 263 eligible patients were enrolled in this study. The median interval between examinations was 441 days (interquartile range, 407–530 days). Patient characteristics are shown in Table 1. The mean age was 69 years, and 30% of patients were aged ≥75 years. Of the patients, 69% were male. Details of bowel preparation and TCS procedures are summarized in Table 2. The mean ingested volume of an OSS was 636 mL, and the total fluid volume was 2049 mL. No patient ingested the preparation incorrectly. Overall, 38% of the patients completed bowel preparation with a single bottle, and only one patient required additional PEG administration. Adverse events occurred in 17% of patients; nausea was the most common adverse event (10%). All events were mild and required no therapeutic intervention. Adequate bowel preparation, defined as BBPS ≥ 6, was achieved in 98% of cases. Figure 1 shows the questionnaire results. Favorable taste acceptability was reported by 51% of the patients (excellent, *n* = 9; good, *n* = 73; neutral, *n* = 53). A total of 143 patients (54%) preferred an OSS for their next TCS preparation. Table 3 presents factors associated with OSS preference, demonstrating that favorable taste acceptability was an independent relevant factor (odds ratio [OR], 4.88). As shown in Table 4, favorable taste acceptability tended to be associated with age ≥ 75 years (OR, 1.69; *p* = 0.07) and male sex (OR, 1.58; *p* = 0.09). Favorable taste acceptability across four groups stratified by age (≥75 or <75 years) and sex is illustrated in Figure 2. Older male patients reported significantly higher rates of favorable taste acceptability compared with younger female patients (63% vs 40%, p = 0.03). Table 5 compares outcomes between the elderly group (age ≥75 years) and the non‐elderly group (age <75 years). The mean ingested volume of OSS did not differ significantly between the two groups (618 vs. 644 mL, *p* = 0.26). However, the total ingested fluid volume was significantly lower in the elderly group (1933 vs. 2098 mL, *p* = 0.04). The proportion of patients achieving adequate bowel preparation (BBPS ≥ 6) was similarly high in both groups (99% vs. 97%, *p* = 0.26). Notably, the incidence of adverse events during bowel preparation was significantly lower in the elderly group compared with the non‐elderly group (8% vs. 21%, *p* < 0.01).

**TABLE 1:** Patients’ characteristics.

	All patients (*n* = 263)
Age	
Mean ± SD (range), years	69 ± 9 (32–89)
≧ 75 years, *n* (%)	78 (30)
Gender, *n* (%)	
Male:Female	181:82 (69:31)
Body mass index	
mean ± SD (range), kg/m^2^	24.2 ± 3.3 (21.9–26.3)
≧ 25	91 (35)
Comorbidity, *n* (%)	
Hypertension	104 (40)
Cerebrovascular disease	7 (3)
Chronic kidney disease	2 (1)
Diverticulum, *n* (%)	
Yes	94 (36)
History of abdominal surgery, *n* (%)	
Yes*	81 (31)
Colorectal surgery	10 (4)
Non‐colorectal surgery	32 (12)
Appendectomy	40 (15)
Reason for examination*	
Surveillance	252 (96)
Screening	5 (2)
Positive FIT	4 (2)
Symptom evaluation	2 (1)

*Not mutually exclusive.

SD: standard deviation.

FIT: fecal immunochemical test.

**TABLE 2:** Details of bowel preparation and total colonoscopy (TCS) procedures.

	All patients (*n* = 263)
OSS volume	
Mean ± SD (range), mL	636 ± 171 (240–960)
≦ 1 bottle, *n* (%)	101 (38)
Water volume	
Mean ± SD (range), mL	1413 ± 468 (500–3000)
Total volume	
Mean ± SD (range), mL	2049 ± 598 (980–3960)
Additional PEG preparation	
Yes, *n* (%)	1 (0.4)
PEG volume, mL	600
Adverse events, *n* (%)	
All*	45 (17)
Nausea	25 (10)
Abdominal bloating	15 (6)
Abdominal pain	8 (3)
Vomiting	5 (2)
BBPS score, *n* (%)	
≧ 6, *n* (%)	257 (98)
Median (range)	
Total	9 (3–9)
Right colon	3 (1–3)
Transverse colon	3 (1–3)
Left colon	3 (1–3)
Adenoma detection, *n* (%)	218 (83)
Cecal or anastomotic intubation, *n* (%)	263 (100)
Insertion time (min), mean ± SD	7 ± 5
Withdraw time (min), mean ± SD	12 ± 6
*Not mutually exclusive	
OSS: oral sulfate solution	
SD: standard deviation	
PEG: polyethylene glycol	
BBPS: Boston Bowel Preparation Scale	

**TABLE 3:** Factors associated with oral sulfate solution (OSS) preference.

Variable	*n* (%)	Univariate OR	95% CI	*p*‐Value	Multivariate OR	95% CI	*p*‐Value
Age ≥75 years	78 (30)	1.12	0.66–1.92	0.67	1.15	0.62–2.13	0.66
Male sex	181 (69)	0.9	0.54–1.53	0.71	0.74	0.41–1.34	0.32
Favorable taste acceptability	135 (51)	4.89	2.89–8.25	< 0.01	4.88	2.84–8.41	< 0.01
Diveculum: no	169 (64)	1.72	1.03–2.86	0.04	1.67	0.95–2.94	0.08
Preparation within one bottle of OSS	101 (38)	1.01	0.61–1.66	0.98	—	—	—
Abdominal surgery history: no	182 (69)	1.54	0.91–2.61	0.11	1.56	0.85–2.87	0.15
BMI ≥ 25	91 (35)	0.74	0.44–1.23	0.24	—	—	—
Hypertension: yes	104 (40)	1.25	0.76–2.05	0.38	—	—	—
Cerebrovascular disease: yes	7 (3)	0.33	0.06–1.71	0.19	0.51	0.09–2.99	0.46
Chronic kidney disease: yes	2 (1)	< 0.01	NA	0.98	—	—	—

BMI: body mass index; CI: confidence interval; NA: not available; OR: odds ratio; OSS: oral sulfate solution.

**TABLE 4:** Factors associated with favorable taste acceptability for an oral sulfate solution (OSS).

Variable	*n* (%)	Univariate OR	95% CI	*p*‐Value	Multivariate OR	95% CI	*p*‐Value
Age ≥75 years	78 (30)	1.44	0.84–2.45	0.18	1.69	0.96–2.99	0.07
Male sex	181 (69)	1.66	0.98–2.81	0.06	1.58	0.92–2.71	0.09
Diveculum: no	169 (64)	1.16	0.70–1.92	0.56	—	—	—
Preparation within 1 bottle of OSS	101 (38)	1.15	0.70–1.89	0.59	—	—	—
Abdominal surgery history: no	182 (69)	1.49	0.88–2.52	0.14	1.6	0.91–2.82	0.1
BMI ≥ 25	91 (35)	0.89	0.54–1.48	0.66	—	—	—
Hypertension: yes	104 (40)	0.98	0.60–1.60	0.92	—	—	—
Cerebrovascular disease: yes	7 (3)	0.37	0.07–1.94	0.24	—	—	—
Chronic kidney disease: yes	2 (1)	< 0.01	NA	0.98	—	—	—

BMI: body mass index; CI: confidence interval; NA: not available; OR: odds ratio; OSS: oral sulfate solution.

**TABLE 5:** Comparison of clinical characteristics and bowel preparation outcomes between elderly (≥75 years) and non‐elderly.

	Elderly (*n* = 78)	Non‐elderly (*n* = 185)	*p*‐Value
Clinical characteristics			
Age			
Mean ± SD (range), years	78 ± 3 (75–89)	65 ± 8 (32–74)	<.01
Gender, *n* (%)			
Male:Female	51:27 (65:35)	130:55 (70:30)	0.47
OSS volume			
Mean ± SD (range), mL	618 ± 169 (240–960)	644 ± 172 (260–960)	0.26
≦ 1 bottle, *n* (%)	31 (40)	70 (38)	
Water volume			
Mean ± SD (range), mL	1316 ± 417 (500–3000)	1454 ± 484 (600–3000)	0.03
Total volume			
Mean ± SD (range), mL	1933 ± 560 (980–3960)	2098 ± 608 (1080–3960)	0.04
BBPS score			
≧ 6, *n* (%)	77 (99)	180 (97)	0.67
Adverse events			
All*	6 (8)	39 (21)	<.01
Nausea	2 (3)	23 (12)	0.02
Abdominal bloating	3 (4)	12 (6)	0.58
Abdominal pain	2 (3)	6 (3)	0.92
Vomiting	1 (1)	4 (2)	0.99

*Not mutually exclusive.

SD: standard deviation.

OSS: oral sulfate solution.

BBPS: Boston Bowel Preparation Scale.

## 4 Discussion

A meta‐analysis of RCTs comparing OSSs with PEG showed that OSSs were significantly superior in terms of bowel cleansing efficacy (mean difference in BBPS score, 0.31) and ADR (risk ratio, 1.13) [7]. In addition, a Japanese RCT comparing an OSS with PEG‐ASC has shown that the mean total volume of an OSS (794.8 mL) is approximately half that of PEG‐ASC (1656.8 mL) [5]. The mean intake volume of an OSS in the present study was further reduced to 636 mL, facilitated by the supplementary administration of sodium picosulfate at bedtime the day before the procedure. Yoshida et al. have reported that effective cleansing can be achieved with sodium picosulfate and 480 mL of OSS (one bottle) in patients with insufficient preparation using conventional methods [11]. However, because the present protocol allowed patients to consume up to 960 mL of OSS (two bottles) and the proportion of elderly patients (≥75 years) was relatively low, only 38% completed preparation with a single bottle, which is lower than previously reported. Nevertheless, overall cleansing efficacy was excellent with 98% of patients achieving BBPS ≥ 6 and a median BBPS of 9.

Patient acceptance of bowel preparation agents is generally influenced by intake volume and taste [12, 13, 14]. In this study, favorable taste acceptability was an independent factor of preference for OSS (OR, 4.88), whereas completion with a single bottle was not significant. These findings suggest that, for an OSS, taste perception may play a more critical role in compliance than intake volume. Taste perception differs by sex and declines with aging [15, 16]. Women tend to prefer sweet tastes, whereas men are more likely to favor bitter or salty tastes. Consistent with these characteristics, favorable taste acceptability for an OSS, which has a distinctive bitter flavor, tended to be associated with an older age (≥75 years; OR, 1.69; *p* = 0.07) and male sex (OR, 1.58; *p* = 0.07). However, palatability remains a concern. Previous reports have indicated that flavor adjuncts or concomitant intake of sports drinks or green tea may enhance palatability and improve adherence [12, 17, 18]. Since OSSs require ingestion of clear fluids in a volume approximately twice that of the solution itself, optimizing accompanying beverages may be particularly important. Such strategies could enhance compliance and improve preparation quality, representing an important avenue for future research.

With the global trend toward population aging [19], the number of elderly patients undergoing TCS is increasing [20]. Using 75 years as the cutoff, two notable strengths of OSSs for elderly patients were revealed in this study. First, although the mean OSS intake volume did not differ significantly between age groups, favorable taste acceptability among elderly patients suggests a lower perceived burden. Despite significantly lower total fluid intake in the elderly group, BBPS scores were comparable, indicating satisfactory cleansing in both groups. Thus, OSSs may improve acceptability while maintaining high‐quality cleansing in elderly patients. Second, adverse events were significantly less frequent in elderly patients. Nausea, the most common adverse event of OSS, occurred in 10% of the patients in this study, compared with approximately 19% in previous studies [7]. Although adverse events are generally more frequent in elderly patients [21], the incidence was lower in this group, largely due to reduced nausea. Other adverse events did not differ significantly between groups. Nausea is an important factor influencing compliance, and bitterness has been reported to enhance nausea [22, 23]. Individuals with higher sensitivity to bitterness are more susceptible to nausea [24]. Since taste sensitivity declines with age, the lower incidence of nausea in elderly patients may be explained by age‐related reductions in taste sensitivity. From the perspective of adverse events, OSSs can therefore be considered a preparation with favorable acceptability, particularly in elderly patients. In Japan, OSS is available as a 480 mL bottled solution that does not require compounding, making it convenient for elderly patients. Moreover, Yoshida et al. have reported that the time required for medical staff to explain this regimen is significantly shorter than for other preparations [11], suggesting advantages for both patients and staff.

This study has several limitations. First, although this was a prospective study, it was conducted at a single center with a relatively small number of Japanese participants, which may introduce bias related to race, ethnicity, or geographic differences and may have influenced taste perception and acceptability. Second, this study included only patients who had undergone outpatient colonoscopy with PEG‐ASC within the previous 2 years, which may have introduced selection bias toward patients with higher acceptability to bowel preparation. In addition, as this was not a head‐to‐head comparison between OSS and PEG‐ASC, the questionnaire‐based assessment may be subject to recall bias. Third, adverse events requiring objective evaluation, such as electrolyte abnormalities or dehydration, were not assessed. Fourth, assessment of adequate bowel preparation relied on patient or caregiver judgment, which may have affected the volume of bowel preparation agent intake. Finally, patients received 75 mg of sodium picosulfate the day before TCS, which may have influenced preparation quality.

## 5 Conclusion

OSS provided high‐quality bowel cleansing despite its low ingestion volume. The regimen was particularly effective for elderly patients, who demonstrated favorable taste acceptability and a low incidence of adverse events. With the growing elderly population, OSSs may be a valuable bowel preparation option that enhances patient acceptability through simple and safe administration.

## Author Contributions

**Investigation**: Taku Yamagata, Takeshi Shimizu, Kai Korekawa, Hiroki Sato, Yutaka Eto, Yosei Sawai, Teruyuki Numata, and Tomohiro Shimada. **Writing – original draft**: Tomohiro Shimada. **Conceptualization**: Tomohiro Shimada, Taku Yamagata, and Yoshihide Kanno. **Methodology**: Tomohiro Shimada, Taku Yamagata, and Yoshihide Kanno. **Writing – review & editing**: Taku Yamagata, Yoshihide Kanno, and Kei Ito.

## Conflicts of Interest

The authors declare no conflicts of interest.

## Funding

The authors declared that this study has received no financial support. The authors have nothing to report.

## Ethics Statement

**Institutional Review Board Approval**: This single‐center, prospective study was approved by the Sendai City Medical Center Ethics Committee (approval No. 2023‐0060) in accordance with the Declaration of Helsinki.

## Consent

This prospective observational study did not involve identifiable personal information. All participants received a written explanation of the study and provided written informed consent prior to enrollment.
